# Trade/Commerce: NAFTA Worries in Juárez

**DOI:** 10.1289/ehp.112-a985a

**Published:** 2004-12

**Authors:** Richard Dahl

In June 2004, Mexico’s government enacted a law requiring industrial facilities to measure, record, and report emissions of 104 chemicals that formerly were subject only to voluntary reporting. Mexico now joins the United States and Canada in mandating public access to such information through databases and other means. Although earlier Mexican laws had established some voluntary reporting, implementation was weak. The new law marks a watershed step in Mexico’s environmental legislation.

Critics say the list of 104 chemicals is paltry compared to the 650 and 268 tracked by the United States and Canada, respectively. But Paul J. Miller, air quality program coordinator at the Montréal-based Commission for Environmental Cooperation (CEC), says what’s most important at this point is to establish a workable rule, then add more substances to the list. The CEC is an environmental research organization created by a side agreement to the North American Free Trade Agreement (NAFTA).

The question is, will those additions come in time? Miller says that health problems related to pollution from heavily traveled border crossings are acute in Mexico because most crossings are located in thickly populated areas. With NAFTA’s 1993 opening of cross-border trading, motorized traffic across the U.S.–Mexico border increased. Heightened security following the terrorist attacks of 11 September 2001 means it takes even longer to get through border checkpoints, resulting in long lines of idling trucks and other vehicles.

Research by Mexico’s National Institute of Public Health concluded that air pollution from high-traffic border crossings between the United States and Mexico poses serious health risks to the children who live near those thoroughfares. In the study, written up in a November 2003 CEC working paper, the researchers examined the effects of air pollutants and ozone on the respiratory health of children in the Mexican city of Ciudad Juárez, which lies just across the border from El Paso, Texas. They found that the traffic pollution–related risks to children were “significant,” and recommended the implementation of cost-effective interventions to reduce the problem.

The researchers studied children’s respiratory health between 1997 and 2001 by matching hospital admission data to each child’s place of residence. They found a connection between poor air quality along the Ciudad Juárez thoroughfare and emergency room visits by children suffering from respiratory illness. Among the poorest citizens, exposure to particulate matter (PM) was related to an increase in infant mortality. Among infants aged 1–12 months, an increase of 20 micrograms PM per cubic meter air on the previous day was associated with a 62% increase in respiratory mortality. If elevated PM was observed on the two previous days, the risk of death was increased by 82%.

Carlos A. Rincón, a scientist with the nonprofit Environmental Defense in El Paso, says the population of Ciudad Juárez has been growing at 4.3% annually for years, mostly due to workers moving there to take jobs created largely by NAFTA. “The border areas should benefit from some of the wealth created by the NAFTA trade,” he says, adding, “Common problems require common solutions.”

Miller points out that NAFTA has no obligations to reduce air pollution, whether it be in Mexico, the United States, or Canada. So the CEC can only issue a call for action, as it did in the Ciudud Juárez working paper.

Fernando Holguin, formerly of the Mexican National Institute of Public Health now working for the Centers for Disease Control and Prevention and Emory University, hopes to enlist the help of municipal authorities in Ciudad Juárez in finding solutions. “The goal would be to either divert traffic flows from some areas where we’ve shown that schools are sensitive to traffic-related emissions or change the time in which certain kinds of vehicles are allowed to travel in certain parts of the city,” he says.

On a broader level, Miller suggests Mexico should reduce the sulfur content in diesel fuel sold there, as the United States and Canada have, and require particle traps on diesel exhausts. Long-term measures would include more expensive responses, such as moving congested border crossings to locations outside of heavily populated areas. In the meantime, Mexican officials plan to start the first phase of mandatory industry reporting by the end of 2004.

## Figures and Tables

**Figure:**
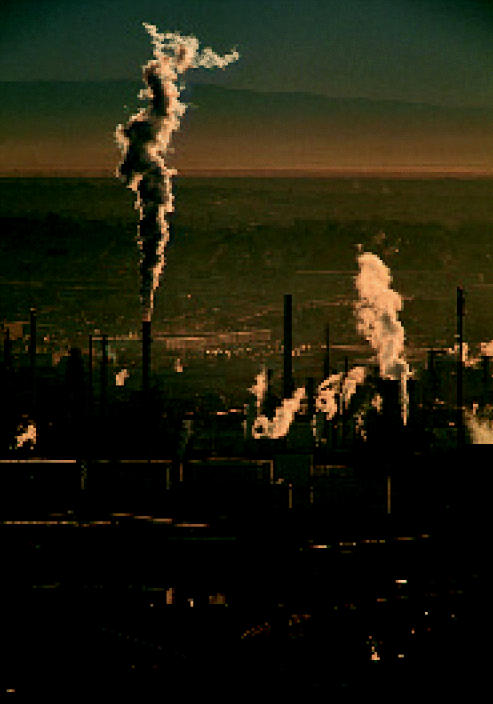
**Ciudad Juárez.** Trading on the environment?

